# Magnon-Optic Effects with Spin-Wave Leaky Modes: Tunable Goos-Hänchen Shift and Wood’s Anomaly

**DOI:** 10.1021/acs.nanolett.3c01592

**Published:** 2023-07-31

**Authors:** Krzysztof Sobucki, Wojciech Śmigaj, Piotr Graczyk, Maciej Krawczyk, Paweł Gruszecki

**Affiliations:** † Institute of Spintronics and Quantum Information, Faculty of Physics, Adam Mickiewicz University, Uniwersytetu Poznańskiego 2, 61-614 Poznań, Poland; ‡ Met Office, FitzRoy Road, Exeter, EX1 3PB, U.K.; § Institute of Molecular Physics, Polish Academy of Sciences, Mariana Smoluchowskiego 17, 60-179 Poznań, Poland

**Keywords:** spin waves, magnonics, magnon-optics, leaky modes, Goos-Hänchen effect, Wood’s anomaly

## Abstract

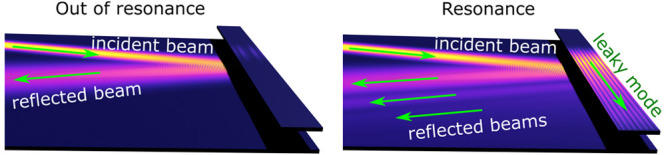

We demonstrate numerically how a spin wave (SW) beam obliquely incident on the edge of a thin film placed below a ferromagnetic stripe can excite leaky SWs guided along the stripe. During propagation, leaky waves emit energy back into the layer in the form of plane waves and several laterally shifted parallel SW beams. This resonance excitation, combined with interference effects of the reflected and re-emitted waves, results in the magnonic Wood’s anomaly and a significant increase of the Goos-Hänchen shift magnitude. This yields a unique platform to control SW reflection and transdimensional magnonic router that can transfer SWs from a 2D platform into a 1D guided mode.

In wave physics, extended and bound modes can be recognized due to their amplitude spatial distribution. The most common are the extended states, which propagate freely in a system. Examples of the second type, which do not necessarily require a structural constraint, include bound states in the continuum (BICs) and leaky modes (LMs).^1^ BIC is a state that exists in the continuous part of the spectrum but is perfectly localized. It was predicted by von Neumann and Wigner for electron waves^2^ and later experimentally observed for photons^3−5^ and phonons.^6,7^ LMs are another type of mode, which are localized but can store energy only for a limited time due to their coupling with extended states. Therefore, LMs can be excited by propagating modes and leak energy into them. Hence, the LM wavenumber is complex, and its imaginary part expresses the rate of energy leakage.^8−10^ The LMs facilitate the occurrence of Wood’s anomaly, which manifests itself as a decrease in the amplitude of reflected waves and is caused by the excitation of an evanescent wave at the interface with some element. It was first reported for light reflected from a grating.^10−12^

An intriguing wave type is the spin wave (SW), that is, a collective precessional disturbance of magnetization in magnetic materials, which is believed to be a promising candidate for information carriers in beyond-CMOS devices.^13−16^ SW optics is more complex than its electromagnetic counterpart and rich in optical phenomena.^17−25^ Many effects from photonics have already been transferred to magnonics, for instance, negative refraction,^26^ anomalous refraction,^25^ graded refractive index effects,^27−29^ and the Goos-Hänchen (GH) effect,^30^ i.e., the lateral shift of the waves’ reflection point at an interface.^31−35^ While the GH effect has been predicted theoretically, it has not yet been experimentally observed for SW beams. Also, the BICs,^36^ LMs, Wood’s anomalies, and resonance effects widely explored in photonics^37^ remain poorly investigated in magnonics.

The use of nanoresonators in the form of ferromagnetic stripes placed over a thin film to modulate SWs has recently attracted attention.^38−50^ In this letter, we numerically study the oblique reflection of a SW beam from the edge of a ferromagnetic film ending with a resonant stripe element^45,46^ [see Figure 1(a)]. We find that a SW beam can excite an LM when resonance conditions are met. The LM emits SWs back into the film while propagating along the stripe. As a result, we observe a decrease in the amplitude of reflected waves, which we interpret as a magnonic counterpart of the Wood’s anomaly. Moreover, we detect multiple reflected beams with tunable positions. Thus, the excitation of LMs allows tuning of the GH shift by several wavelengths. Our results open a route for the exploitation of the demonstrated effects in various magnonic applications, including designing resonant SW metasurfaces in planar structures suitable for integration with magnonic devices, converting extended SWs into waves guided along a stripe and exploiting the third dimension in integrated magnonic systems.

**Figure 1:**
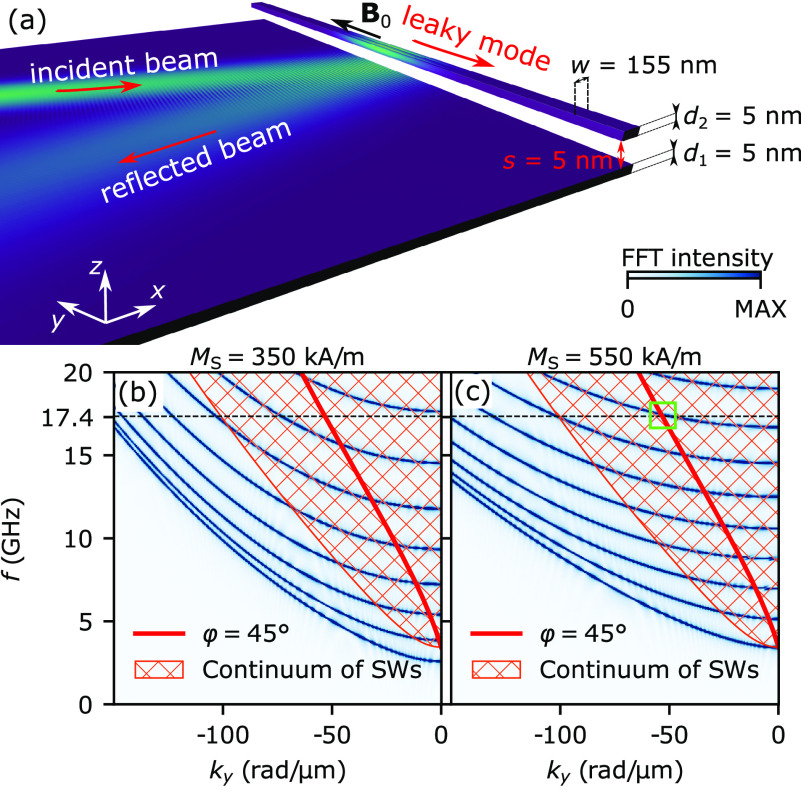
(a) Geometry of the system. Color represents the SW amplitude and indicates the incident and reflected SW beams in the film as well as the LM in the stripe. (b,c) Dispersion relation of SWs. The blue colormaps in the background represent dispersion relations of the resonant-stripe element of the SWs propagating along the stripe with (b) *M*_S_ = 350 kA/m and (c) *M*_S_ = 550 kA/m. The hatched region displays the continuum of SWs in the CoFeB layer. The thick red line represents the analytical dispersion relation of SWs propagating in the film at an angle of φ = 45° to **B**_0_. The horizontal black dashed line indicates the frequency *f*_0_ = 17.4 GHz used in the steady-state simulations. The green square in (c) shows the crossing at *f*_0_ = 17.4 GHz of the beam and stripe dispersion relations.

We consider a half-infinite CoFeB layer of thickness *d*_1_ = 5 nm with a saturation magnetization of 1200 kA/m and exchange constant of 15 pJ/m. Above the film lies a ferromagnetic stripe of width *w* = 155 nm and thickness *d*_2_ = 5 nm aligned with the layer’s edge. We assume that the exchange constant in the stripe equals 3.7 pJ/m, and the value of its saturation magnetization *M*_S_ varies. Both elements are separated by a dielectric nonmagnetic layer of thickness *s* = 5 nm; see Figure 1(a). We will refer to the stripe and the layer directly below it as the bilayer. The system is uniformly magnetized by an external magnetic field *B*_0_ = μ_0_*H*_0_ = 0.01 T directed along the stripe (the *y*-axis). We set the damping parameter α to 0.0004 and the gyromagnetic ratio γ to −176 rad GHz/T. At normal SW incidence, this geometry is a magnonic realization of the Gires–Tournois interferometer, offering multiple Fabry–Pérot resonances.^45^ We analyze the oblique incidence of a 775-nm-wide SW beam at the frequency *f*_0_ = 17.4 GHz (wavelength λ = 103 nm) and at an angle of 45° (angle of the phase velocity with respect to the *x*-axis). We employ MuMax3^51^ to perform micromagnetic simulations of the dynamics of the magnetization **m**(**r**,*t*) in the system (for more details see Supporting Information).

The dispersion relations of SWs propagating along the stripe placed above the layer for two selected values of the stripe magnetization *M*_S_, i.e., 350 kA/m and 550 kA/m, are shown by the blue colormaps in Figure 1(b) and (c), respectively. The hatched area is the continuum spectrum of propagating SWs in the CoFeB film, calculated analytically,^52^ and the red line is the dispersion of the SW beam (see Supporting Information). For *M*_S_ = 550 kA/m, the analytical dispersion crosses the bilayer dispersion at the frequency of the SW beam *f*_0_ = 17.4 GHz; see the green square in Figure 1(c). Here, the wavevector component *k*_*y*_ of the incident wave matches the wavenumber of a stripe mode; therefore, we expect the incident SW beam to excite that mode. For *M*_S_ = 350 kA/m, there is no phase matching at *f*_0_ = 17.4 GHz; thus, the coupling between the incident SW and stripe modes is suppressed.

To verify our predictions, we examine the reflection of the SW beam from the bilayer edge for the two considered values of *M*_S_ in the stripe. In the simulations, we use a continuous excitation of the SW beam and analyze the linear response for the steady-state SW distribution in the system (see Supporting Information). Figure 2 presents the steady-state |*m*_*x*_| amplitude distributions for the stripe magnetizations *M*_S_ = 350 and 550 kA/m, respectively.

**Figure 2:**
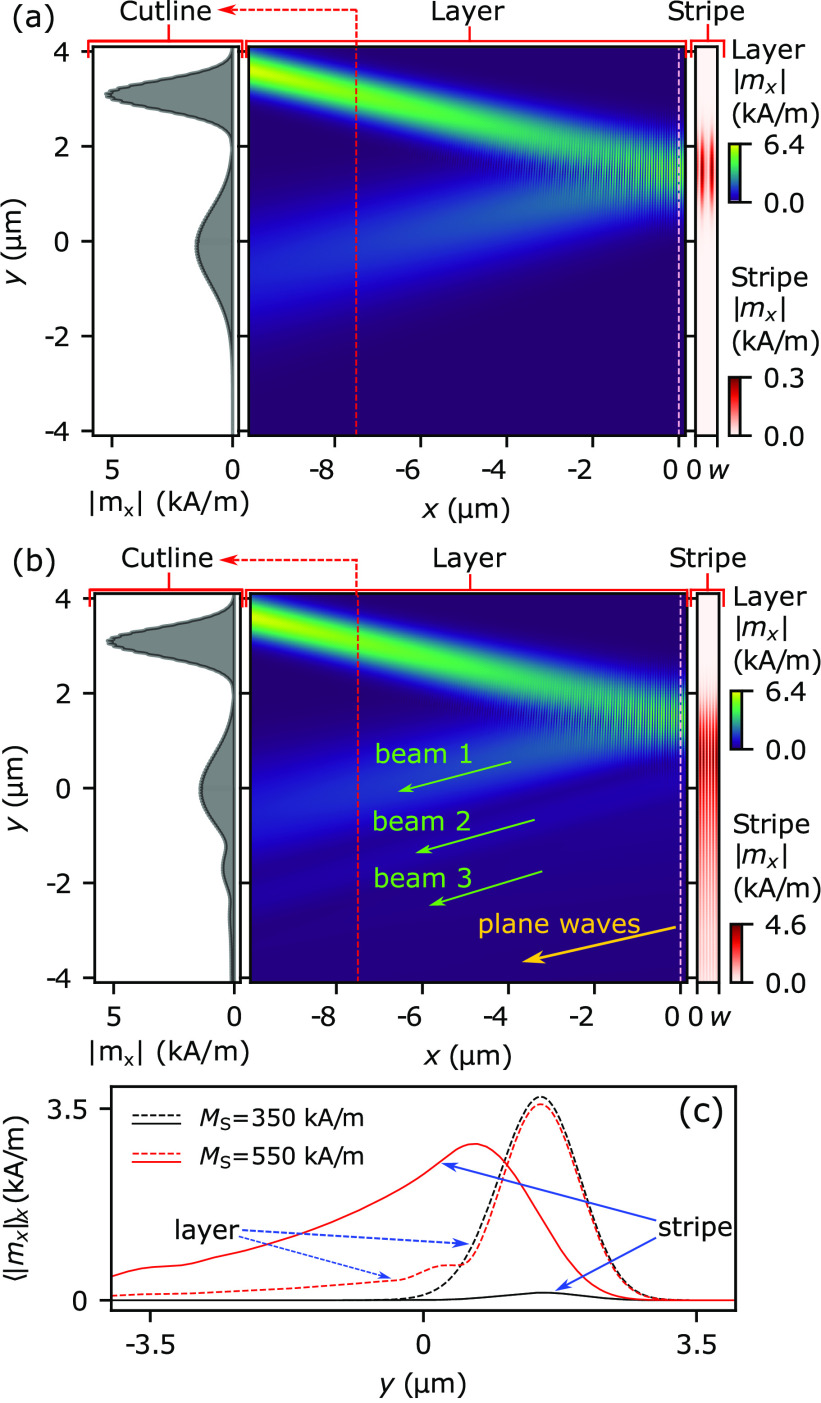
(a,b) Colormaps of the SW amplitude distribution of magnetization’s in-plane dynamic component |*m*_*x*_| . Middle panels show |*m*_*x*_| in the CoFeB layer. The colormaps in the bars on the right present |*m*_*x*_| in the stripe. The panels on the left show |*m*_*x*_| in the layer in a cutline through the layer at the position *x* = −7.5 μm indicated with a red-dashed line in the central panel. Results for the system with a stripe of (a) *M*_S_ = 350 kA/m and (b) *M*_S_ = 550 kA/m. (c) Averaged  as a function of *y* in the stripe (solid lines) and in the layer directly under the stripe (dashed lines). The black and red lines represent results for the system with *M*_S_ = 350 kA/m and *M*_S_ = 550 kA/m, respectively.

In the case of *M*_S_ = 350 kA/m [Figure 2(a)], the SWs are excited, but oscillations are present only in the region directly above the incident spot. Their amplitude is an order of magnitude smaller than in the layer. In the far field, we see only a single reflected SW beam (cf. the left panel in Figure 2(a)). Both the incident and reflected beams have Gaussian envelopes with an apparent increase in width due to beam divergence.

We observe a different behavior in the case of *M*_S_ = 550 kA/m. First, the SW amplitude in the stripe is comparable with that of the SW beam in the layer. Moreover, we observe the propagation of SWs along the stripe in the direction opposite to the *y*-axis, which is consistent with the group velocity direction extracted from the dispersion relation for SWs in the stripe. The mode in the bilayer emits SWs back to the layer during its propagation, which is a clear indication of its LM nature. Furthermore, we observe the formation of new SW beams in the layer that are parallel to the primary beam (see Figure 2(b)). Two new beams are clearly visible in the left panel, which shows the SW intensity cross-section taken at *x* = −7.5 μm. Note that there are also plane waves propagating outward from the interface.

Let us examine the change in the SW amplitude as it propagates along the bilayer. In Figure 2(c) we see a significant difference between the SW modes in the two considered cases. As expected, for the stripe with *M*_S_ = 350 kA/m, the SW amplitude in the stripe is negligible. However, when the phase-matching condition is fulfilled at *M*_S_ = 550 kA/m, we see an efficient excitation of the LM that propagates in the −*y*-direction (see the solid red line in Figure 2(c)). Note that the distribution of the amplitude ⟨|*m*_*x*_|⟩_*x*_ in the layer below the resonator along the *y*-axis can be decomposed into several superimposed Gaussian functions (discussed in detail in the following paragraph), namely, a dominant one associated with the primary reflected SW beam and several additional ones with smaller amplitudes (see the dashed red line). This opens channels for energy leakage from the LM to the film.

To gain a deeper insight into how LM emission occurs over time, we perform simulations of a SW packet to complement the steady-state simulations discussed previously (see also Supporting Information). The full width at half-maximum (FWHM) of the packet is 0.5 ns and the angle of incidence is 45°. In Figure 3, we present two snapshots of the reflected wavepacket from the simulation with the stripe magnetization *M*_S_ = 550 kA/m (the video in Supporting Information, Movie S5). These simulations confirm that the leaky mode excited by the incident SWs propagates along the bilayer and reemits SWs in the form of plane waves without a constant supply of SWs from the incident SW beam. In addition, Movie S5 shows that the amplitude of the excited mode in the stripe bounces obliquely between the edges of the stripe (this is apparent especially at lower SW amplitudes). We stipulate that this bouncing in the stripe is the source of a spatial shift of the third and next reflected beams. The bouncing mode re-emits its energy at a higher rate when it reflects from the left edge of the resonator, giving rise to new reflected beams in the system.

**Figure 3:**
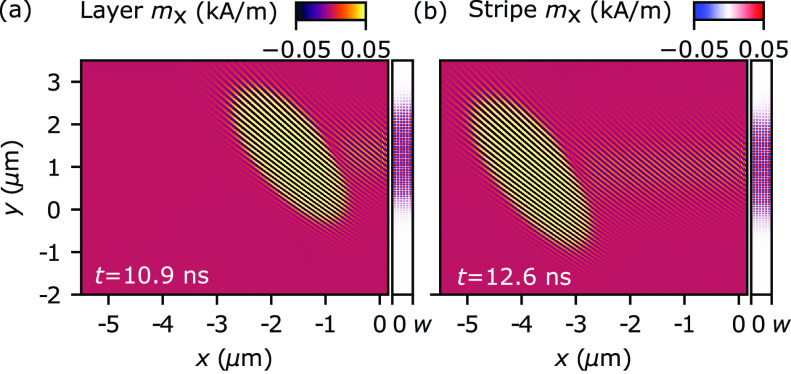
Reflection of the SW wavepacket on the bilayer resonant-stripe element (*M*_S_ = 550 kA/m) for (a) *t* = 10.9 ns and (b) *t* = 12.6 ns. Note that the amplitudes of SWs are amplified to better visualize SWs reemited by a leaky mode in the stripe; see the colorbars.

To understand the formation of multiple reflected beams at resonance [Figure 2 (b)] we perform the analysis proposed by Tamir and Bertoni^10^ to explain the formation of an additional reflected beam in the case of electromagnetic waves. The reflectance coefficient of the incident SW beam at an interface with the LM can be described as ρ(*k*_*y*_) = *e*^*i*Δ^(*k*_*y*_ – *k*_p_^*^)/(*k*_*y*_ – *k*_p_), where Δ is the phase shift between the incident and reflected beams; *k*_*y*_ is the tangential component of the incident wavevector; and *k*_p_ = κ + *iν* is the LM wavenumber. Because of the tangential component conservation rule, both *k*_*y*_ and *k*_p_ have the same direction of propagation with respect to the *y*-axis. As the Tamir–Bertoni model shows, when the stripe mode has a bound (non-leaky) character, i.e., ν = 0, the LM is not excited by the incident beam; thus, it has no effect on the reflected beam. Excitation of the stripe mode occurs when it takes on a leaky character and becomes more effective as the imaginary component ν increases. Concurrently, an increase in ν accelerates the transfer of energy back into the layer; hence, at a specific value of ν, the secondary beam overshadows the main reflected beam. This is qualitatively reflected in our simulation results (Supporting Information). However, our simulations show the presence of several reflected beams instead of two, as in the Tamir–Bertoni model. This discrepancy may be due to some differences between the Tamir–Bertoni model and our system, such as neglecting the higher-order poles of the reflection coefficient in the Tamir–Bertoni model, the finite stripe width, and bouncing of the SW amplitude between the edges of the stripe described in the previous paragraph.

Let us examine how the reflection is affected by the change of the stripe’s *M*_S_ while going through resonance. Figure 4(a) illustrates the dependence of the average SW amplitude within the stripe, ⟨|*m*_*x*_|⟩_stripe_, on *M*_S_ in the range 350–750 kA/m. It shows an increase of the amplitude for *M*_S_ ∈ (450–650) kA/m with a maximum reached at 590 kA/m. Therefore, a band crossing occurs near 17.4 GHz for a wide range of *M*_S_. The origin of this broadband resonance effect together with dispersion relations for various values of *M*_S_ can be found in the Supporting Information.

**Figure 4:**
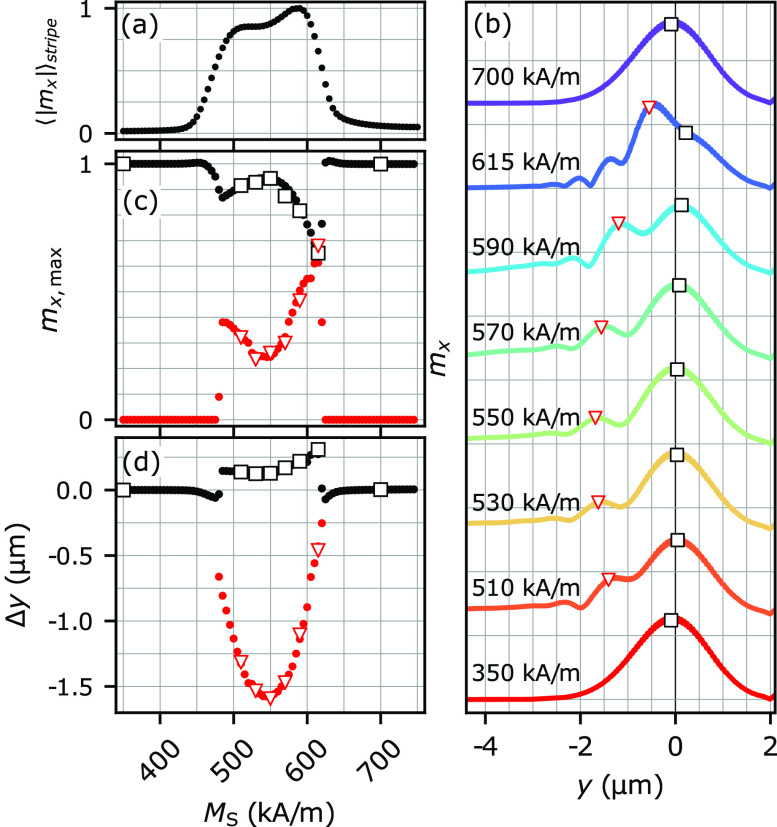
(a) Averaged |*m*_*x*_| over the volume of the stripe as a function of its *M*_S_. (b) Comparison of several reflected beam cutlines at *x* = −7.5 μm for different values of *M*_S_. The maximal amplitudes of the primary and secondary beams are marked with black squares and red triangles, respectively. (c) The maximal amplitudes of the primary and secondary beams in arbitrary units as a function of *M*_S_. (d) The spatial GH shift of the beams’ maxima with respect to the system with *M*_S_ = 350 kA/m as a function of *M*_S_.

In Figure 4(b), we superimpose several cutlines through the layer’s |*m*_*x*_| distributions in the far field for different values of *M*_S_. We quantify the positions and amplitudes of reflected beams by fitting Gaussian curves to the cutlines in the far field; more details are given in Supporting Information. We mark the positions of the maxima of the primary reflected beam with black squares and those of the secondary beam with red triangles. The position *y* = 0 represents the position of the reflected beam for *M*_S_ = 350 kA/m at *x* = −7.5 μm. It is evident that the positions and amplitudes of the reflected beams change with *M*_S_. As we approach the resonance and LM excitation is observed, the primary beam becomes weaker (see Figure 4(c)), its amplitude decreasing by almost 40% at *M*_S_ ≈ 615 kA/m. We interpret this decrease as a magnonic analogue to the Wood’s anomaly^11^ since the amplitude of the reflected beam decreases due to the excitation of the stripe’s localized mode. As the primary beam amplitude decreases, the amplitude of the secondary beam increases, and for *M*_S_ ≈ 615 kA/m, it is even higher than the amplitude of the primary beam. These facts adequately reflect the Tamir–Bertoni analytical model and indicate an increase of the imaginary part of the LM wavenumber, meaning that more energy is leaked by the LM and that the energy of the incident beam is more efficiently directed to the secondary reflected beams.

As shown in Figure 4(d), the reflected beams are shifted with respect to the reference nonresonant scenario, i.e., *M*_S_ = 350 kA/m. This implies the possibility of manipulating the value of the GH shift, which for *M*_S_ = 350 kA/m takes the value of +12 nm, namely, around 10% of the incident SW wavelength. It is a typical value of GH shifts for SWs, which are usually smaller than the SW wavelength.^31,32,34^ The positions of the primary and secondary reflected beams change when LMs are excited (cf. Figures 4(a) and (d)). The primary and secondary beams move toward positive and negative *y*-coordinates, respectively. The displacement of the primary beam ranges from −71 to 300 nm, reaching a maximum at *M*_S_ = 615 kA/m. Therefore, by adding a stripe above the edge, we can profit from the resonance effect to significantly enhance and manipulate the value of the GH shift, reaching the value of up to almost three wavelengths. This is already a measurable value, which is essential for the experimental verification of the GH shift for SW beams. Moreover, the shift of the secondary beam is even larger and reaches up to −1600 nm. Notably, for *M*_S_ ≈ 615 kA/m (the case where the amplitude of the secondary beam is greater than the amplitude of the primary beam), the lateral beam displacement is −456 nm.

In conclusion, we have shown a new way to control SW propagation in a thin ferromagnetic film using a magnonic resonance element formed by depositing a ferromagnetic stripe on top of the film. We found a magnonic counterpart to Wood’s anomaly as well as a GH shift measurable with state-of-the-art experimental techniques. Our results have several important implications for magnonics and its application. First, our system is a platform for studying the controlled reflection and scattering of SWs. Under certain conditions, the incident SW beam can excite an LM in the resonance element, which emits a portion of its energy in the form of new SW beams. Note that the resonance criterion is fulfilled in the system not for a specific *M*_S_ but for quite a broad range of *M*_S_, covering standard values of Py. This indicates the feasibility of an experimental realization of such an interferometer. Moreover, our system allows an easy change of the GH shift magnitude by several wavelengths, up to 450 nm for the primary beam and 1600 nm for the secondary beam. The resonant coupling described in this paper adopts properties from a spectrum between a mode strictly confined to the bilayer and an SW from the continuum of modes in the film. Thus, the same effects are expected for other angles of incidence and other confined modes at different frequencies. Although the analysis in the main part of the paper was done by varying the stripe magnetization *M*_S_, in the Supporting Information we show that similar effects can be observed when the modulated parameter is the frequency or the stripe width, which can be changed more easily in experiments. Moreover, for the near-resonance scenario, the amplitude of the secondary beam can exceed the amplitude of the primary reflected beam to an even greater extent. Finally, the proposed geometry allows transferring the energy of a SW beam propagating in the film to the stripe; i.e., it allows a high-efficiency transfer of SWs from 2D platforms into 1D waveguides, forming a transdimensional magnonic router in a similar manner to the one that was proposed for plasmons.^53^ This is crucial for designing magnonic circuits and exploiting the third dimension for signal processing.

## Supporting Information Available

Description of the numerical methods, the data postprocessing, and the results of additional simulations (PDF)
Video of the calculated steady-state SW intensity distribution as a function of the stripe magnetization *M*_S_ (MP4)
Video of the calculated steady-state SW intensity distribution as a function of the beam frequency (MP4)
Video of the system’s dispersion relation as a function of the stripe magnetization *M*_S_ (MP4)
Video of the wavepacket reflection from an interferometer with stripe magnetization *M*_S_ = 350 kA/m (MP4)
Video of the wavepacket reflection from an interferometer with stripe magnetization *M*_S_ = 550 kA/m (MP4)
