# Supplementary material for: Magnon-Optic Effects with Spin-Wave Leaky Modes: Tunable Goos-Hänchen Shift and Wood’s Anomaly
Source: Nano Lett. 2023 Jul 31;23(15):6979–84. doi: 10.1021/acs.nanolett.3c01592 (PMC10416350; doi:10.1021/acs.nanolett.3c01592)
Supplement: Supplementary file 1 — nl3c01592_si_001.pdf [file nl3c01592_si_001.pdf]

# Magnon-optic effects with spin-wave leaky modes: tunable Goos-Hänchen shift and Wood's anomaly

Krzysztof Sobucki,[1,*] Wojciech Śmigaj,[2] Piotr Graczyk,[3] Maciej Krawczyk,[1] and Paweł Gruszecki[1,†]

[1] *Faculty of Physics, Adam Mickiewicz University, Uniwersytetu Poznańskiego 2, 61-614 Poznań, Poland*
[2] *Met Office, FitzRoy Rd, Exeter, EX1 3PB, UK*
[3] *Institute of Molecular Physics, Polish Academy of Sciences, Mariana Smoluchowskiego 17, 60-179 Poznań, Poland*

## I. SUPPLEMENTARY MATERIALS

### 1. *Numerical methods*

To perform numerical simulations, we employ the open-source environment Mumax3 [1]. This environment solves Landau-Lifshitz-Gilbert equation using the finite-difference method in the time domain. The simulated system has dimensions 12.7 µm, 10.2 µm and 15 nm (along the $x$, $y$, $z$ axis, respectively). We discretize the simulated domain with a regular mesh of unit cell $5 \times 5 \times 5$ nm$^3$ (along the $x$, $y$, $z$ axes). In order to simulate an infinitely long system in $y$-axis and half-infinite system along the $x$-axis, we impose at all edges of the system except the one where the stripe is located, absorbing regions where the damping constant $\alpha$ increases quadratically to the value $\alpha_{\text{edge}} = 0.5$ at length of $L = 625$ nm.

We perform three types of simulations:

- calculations of the dispersion relation of the system for different values of stripe's $M_{\text{S}}$,
- calculations of the steady-state for oblique incidence of continuously emitted spin-wave (SW) beam,
- reflection of a wave-packet with step-by-step observation of SWs reflection from the bilayer interface.

### 2. *Dispersion relation computations*

To accelerate dispersion relation computation for SWs propagating along the stripe, we perform simulations for a narrower system of width 1270 nm along the $x$-axis since, as we verified, it provides exactly the same results as simulations for the system of widths 12.7 µm. In this type of simulations, we place the SW source in the stripe parallel to the $x$-axis in the central part of the stripe. To excite SWs for all wavevectors up to the cut-off wavevector $k_{\text{cut}} = 150$ rad/µm and frequencies up to the cut-off frequency $f_{\text{cut}} = 20$ GHz, we use the following spatial and temporal distribution of the microwave field being linearly polarized along the $z$-axis

$$\begin{aligned} h_z(t;x,y) =& h_0 \text{sinc}(k_{\text{cut}} y) \text{sinc}(2\pi f_{\text{cut}}(t - 8/f_{\text{cut}}) \\ &\times \sum_{n=0}^{N} \left[\cos(2\pi n\ x/w) + \sin(2\pi n x/w)\right], \end{aligned} \tag{S1}$$

where the summation of $n$ is used to increase the efficiency of the higher order modes excitation (we assume $N = 5$). We use the time sampling $t_{\text{sampl}} = (2.2 f_{\text{cut}})^{-1}$ and save first 1000 snapshots of the system's response to the microwave excitation. To obtain the dispersion relation $D(f, k_{\text{y}})$ we employ following formula

$$D(f, k_y) = \langle |F_{t,y}\{m_x(t,y,x)\}| \rangle_{x \in \langle 0, \text{w} \rangle}, \tag{S2}$$

where $F_{t,y}$ is the two-dimensional $(t, y)$ fast Fourier transform (FFT), $m_{\text{x}}(t, y, x)$ is the magnetic response taken only from the stripe. The absolute value of the outcome of FFT $(|F_{t,y}\{m_x(t,y,x)\}|)$ is a space-averaged along resonator's width $x \in \langle 0, w \rangle$ and represents $D(f, k_{\text{y}})$.

* krzsob@st.amu.edu.pl
† gruszecki@amu.edu.pl

The results of the dispersion relation calculations for different values of stripe $M_S$ are compiled into a short video that can be found in supplementary materials, Movie S3. With an increase in the value of $M_S$, the positions and shape of the dispersion relation bands change. For easier analysis, the dashed lines indicate the parameters of the SW beam excited in the simulations. We show in the video that only in a specific range of $M_S$ values, the bands cross the lines that represent the parameters of the incident SW beam. We interpret this range of $M_S$ as a region of efficient SW excitation in the magnetic stripe.

### 3. *Steady-state simulations*

In order to excite SW beam, we use microwave magnetic field located at the left upper quarter of the layer. The spatial distribution of the dynamic magnetic field is in the rotated coordinate system $(x', y')$ by 45° with respect to the $y$-axis. The spatio-temporal function of the dynamic magnetic field is given by a formula

$$\begin{aligned} B_{\text{ext},x}(t, x', y') &= A(1 - e^{-0.2\pi f_0 t})R(x')G(y') \\ &\times[\sin(k_0 x')\sin(2\pi f_0 t) + \cos(k_0 x')\cos(2\pi f_0 t)], \end{aligned} \tag{S3}$$

where $A = 0.1B_0$ is the amplitude of the dynamic field ($B_0$ is the external magnetic field set along the system's $y$-axis of magnitude $B_0 = 0.01$ T), $R(x') = \Theta(-x' + \frac{w_a}{2})\Theta(x' + \frac{w_a}{2})$ is a rectangle function, which describes antenna's shape along its $x'$ coordinate ($\Theta$ is Heaviside step function, antenna's width $w_a = 30$ nm), $G(y') = \exp(-\frac{y'^2}{4\sigma_y^2})$ is a Gaussian function defining antenna's shape along the $y'$-axis ($\sigma_y = 330$ nm), $k_0 = 60.96\ \frac{\text{rad}}{\mu\text{m}}$ is the wavevector and $f_0 = 17.4$ GHz is the frequency of the excited SWs. Eq. (S3) enables unidirectional emission of SWs[2]. We use the antenna to constantly emit the SW beam for 41 ns, after this time the system reaches the steady-state. Subsequently, we store time and space dependence of magnetization distribution for one period of SWs excitation in form of 25 snapshots of magnetization distribution in the system with a sampling interval $1/(25f_0)$.

The stored magnetization dependence on time in the steady-state can be converted into complex SW amplitude distribution at frequency $f_0$. It simplifies the analysis of the SW amplitude and phase. To make such a conversion, we calculate pointwise FFT over time and select results only for $f_0$.

### 4. *Simulations of the reflection of wave-packet*

To simulate the wave-packet reflection, we use the same spatial distribution of the dynamic magnetic field as in Eq. (S3). However, the time dependence of the formula is multiplied by the Gaussian envelope described by the expression $\exp(-(\frac{t}{2\sigma_f})^2)$, with $\sigma_f = 0.05f_0$. It provides the packet with full width at half maximum (FWHM) in the time domain of 0.5 ns. As the result of the simulations, we save 250 snapshots of the propagating wave-packet with a time step of 0.057 ns. The results of simulations with stripe's $M_S = \{350, 550\}$ kA/m are compiled into short movies that can be found in supplementary materials, Movies S4 and S5. In the movie for the $M_S = 350$ kA/m stripe, the packet is reflected from the interface without any substantial excitation of the SWs in the stripe. However, in the movie with the $M_S = 550$ kA/m stripe, the excitation of the SWs in the stripe is evident. The mode formed in the stripe propagates along the stripe, and the re-emission of additional SWs to the layer is visible in the magnification.

### 5. *Influence of the beam width on excitation of modes in the stripe*

We check the influence of the beam width on excitation of the modes in stripe for $M_S = 460$ kA/m as for this value of $M_S$ for a beam with FWHM = 775 nm, used in the main simulations, we observe the beginning of stripe's mode excitation. We perform series of simulations with beam of varying FWHM. In Fig. S1 we show the results of simulations for beams with FWHM = $\{517, 775.5, 1551\}$ nm. In Figs. S1(a,b) we present SW intensity distributions in the layer for two beams with FWHM = 517 nm (a) and FWHM = 1551 nm (b). In Fig. S1(a) with the narrow beam several reflected beams are evident. Their number is bigger than we presented in the main body of the paper. Conversely for a wide beam, as in Fig. S1(b), no reflected beam stratification is visible. For easier analysis in Fig. S1(c) we provide a plot with cutlines through SW intensity distributions for different SW beams, the cutlines are marked with red dashed lines in Figs. S1 (a,b). The result of the narrowest beam is presented with the orange line. In this case the amplitude of the primary beam is the smallest but a phalanx of additional reflected beams are well visible. The results for the widest beam is shown with red line, here the reflected beam has regular Gaussian envelope and no additional reflected beams are visible. The green line presents the results for the beam with FWHM = 775.5 nm,

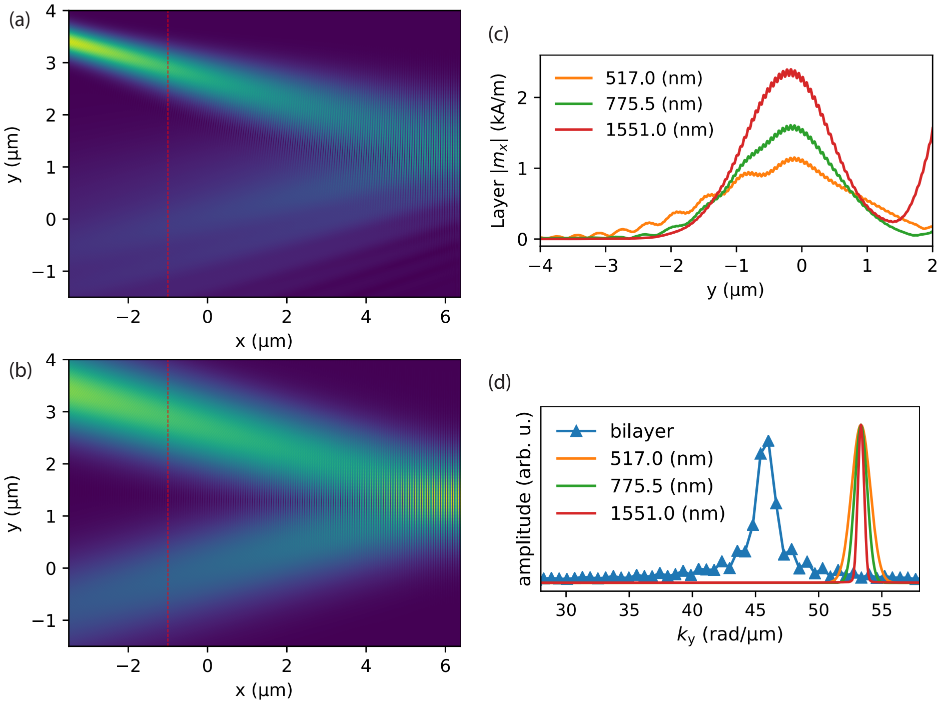


FIG. S1. Beam width sweep. (a) SW intensity distribution in the layer with SW beam with FWHM 517 nm at the antenna. (b) SW intensity distribution in the layer with SW beam with FWHM 1551 nm at the antenna. (c) Comparision between SW intensity cutlines (marked with red dashed lines in (a,b)) for SW beam with FWHM $517, 775.5$ and 1551 nm (orange, green and red lines respectively). The increase of the amplitude for the red line at $y > 1.5$ µm is caused by the widened incident beam. (d) Overlap of the beams width in the inverse space with the system's dispersion relation at $f = 17.4$ GHz (blue line).

used in the main simulations. This case is intermediary between previously presented narrow and wide beams. The secondary reflected beams are present although there are not as distinctive as in the case of the narrowest beam.

We propose following explanation to the fact that narrow SW beam is able to excite the resonator's mode more efficiently than a wide beam. We bind the efficiency of mode excitation with an overlap between the beam's dispersion relation and the bilayer's dispersion relation. In Fig. S1(d) we show the cutlines through bilayer's dispersion relation at frequency $f = 17.4$ GHz and dispersion relations of the beams. The dispersion relations of the beams are presented as Gaussian curves, which centres are calculated from the Kalinikos-Slavin formula [3], and their widths are obtained by calculating beam's widths in the reciprocal space. It is evident that the narrowest beam has the biggest width in the reciprocal space and because of that has the biggest overlap with bilayer's dispersion relation. Such a situation leads to more efficient coupling between the beam and the bilayer than in any other case presented in this analysis. Thus, the narrower beams have possibility to excite resonator's mode more efficiently in our system. The analysis presented here is more qualitative rather than quantitative as the beams widen during their propagation and during reflections have bigger FWHM than at the antenna. Thus the overlap of dispersion relation at the reflection is even smaller than presented in Fig. S1(d). However, the ratio between the overlap and the width of particular beam is the same as presented, so our explanation is justified.

### 6. *Results of comparision with Tamir-Bertoni model*

In this section we compare the results of our simulations with an analytical model proposed by Tamir and Bertoni[4] in more detail. Tamir and Bertoni showed that an incident beam of light is able to excite a leaky mode (LM) at the edge of the system. The excited edge mode propagates along the edge and emits waves back to the system. As our findings are a close analog to Tamir-Bertoni model but in the realm of magnonics, we try to apply Tamir-Bertoni mathematical description to our simulation's results. Tamir and Bertoni proposed the reflectance coefficient in the form of $\rho(k_y) = e^{i\Delta}(k_y - k_\mathrm{p}^*)/(k_y - k_\mathrm{p})$, where $k_\mathrm{p} = \kappa + i\nu$ is a complex wave vector of the LM. They solved the system analytically under assumptions of well collimated beam incident and an angle of perfect coupling between the beam and edge mode. Additionally they also assumed that only the first pole in reflectance coefficient $\rho(k_y)$ provides a substantial input to the calculations. Their formula of the reflected light amplitude has two components which

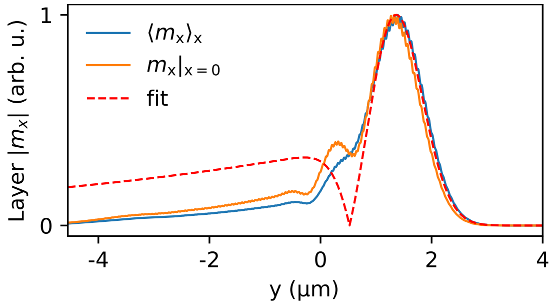


FIG. S2. Comparision between simulation results (blue line mean values of SW intensity in the layer under the stripe, orange line SW intensity cutline in the layer under the left edge of the stripe) and Tamir's model numerical fit (red dashed line) to the SW intensity mean value.

describe the primary $E_0$ and the secondary beam $E_1$

$$\begin{aligned} E_{\text{refl}} &= E_0 + E_1, \\ E_0 &= Ae^{-((y-y_0)/w_b)^2}, \\ E_1 &= -E_0(2 - \pi^{\frac{1}{2}}\nu w_b e^{(\gamma')^2}\text{erfc}(\gamma')), \end{aligned} \tag{S4}$$

where $A$ is the amplitude of the primary beam, $y_0$ is the centre of primary beam, $w_b$ is the width of beam at $\frac{1}{e}$ of its amplitude, $\nu$ is the imaginary part of the LM wave-vector, $\gamma'$ is a new coordinate system defined for the secondary beam as $\gamma' = \frac{\nu w_b}{2} - \frac{y-y_0}{w_b}$ and erfc is the Gauss error function.

In Fig. (S2) we present a numerical fit of Eq. (S4) to the simulation data. The blue line in Fig. (S2) shows the mean value of SW intensity averaged in the volume of the layer directly under the stripe. The dashed red line is the numerical fit to this data. It is evident that the analytical model provided by Tamir and Bertoni agrees only qualitatively with the results of our simulation. Namely, Tamir-Bertoni model describes properly the primary beam in our simulations but fails to precisely fit to the secondary beam. In this case analytical model only indicates separation between the primary and secondary reflected beams. However, it does not recreate the shape of secondary beam, it only shows a long tail of nonzero amplitude left to the primary beam. We see a several reasons why Tamir-Bertoni model does not work properly with our simulation's results. Firstly, Tamir-Bertoni model was developed for the light beam, which physics is governed by Helmholtz equation, while in our case we deal with SWs that are described by Landau-Lifshitz equation. Secondly, in Tamir-Bertoni model the edge of the system is infinitely narrow but in our simulations we regard the bilayer with finite width as an edge. We show the difference between the approach of wide and narrow edges in Fig. (S2). Here, the blue line describes average SW intensity under the stripe and the orange line is a cutline through SW intensity in the layer under the left edge of the stripe. The results of the edge cutline have more distinctive peaks with bigger amplitudes nevetherless in our calculations we have to choose the average values of SW intensity to take into account contribution from whole bilayer width. At last Tamir-Bertoni model is based on several assumptions, such as choosing an optimal incident beam angle to couple with the edge mode, that are not met in our numerical simulations. We did not look for the ideal conditions for the SW beam incident in our simulations as their are impractical in designing experiments to confirm our numerical findings.

### 7. *Influence of stripe's width on reflection*

We explored new resonances cases by performing simulations with fixed material parameters but with varying resonator's width. We chose $M_S = 550$ kA/m value as in the paper's main body which corresponds to the case with the most pronounced spread among the secondary beams. Then we run several simulations with stripes of widths $w$ in a range from 100 nm to 205 nm. In Fig. S3(a) we present the SW intensity density in the stripe as a function of stripe's width (the values in the plot are normalised to the highest value for $w = 155$ nm). In Fig. S3(a) we can see several peaks for certain values of $w = \{110, 135, 155, 175, 200\}$ nm. For each of these cases there is an excitation of LM in the stripe and creation of the secondary beams in the layer below the stripe, Fig. S3(b-d). Thus we prove that the resonance conditions in described system can be also achieved by changing other parameters of the system than its material parameters.

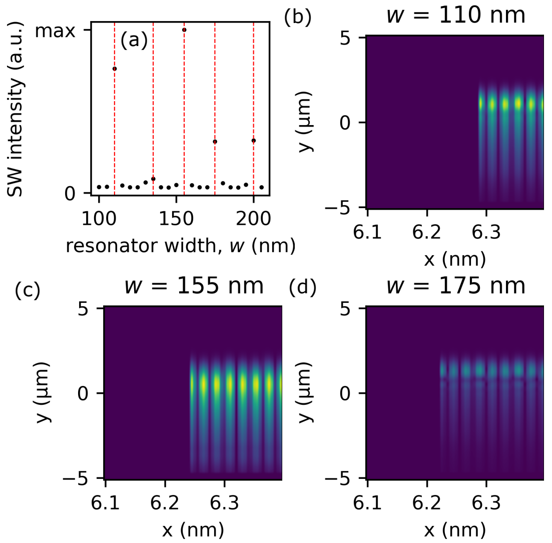


FIG. S3. (a) Intensity of SW in the stripe as a finction of stripe's width. (b-d) Visualisations of the modes excited in the stripe for different values of stripe's width as SW intensity distribution, (b) width 110 nm, (c) width 155 nm, (d) width 175 nm. All (b-d) figures have the same colour scale normalized to the highest value in case 155 nm (c).

### 8. *Influence of SW frequency on reflection*

We obtain parameters of the reflected beams by fitting a sum of Gaussian functions to the cutline through SW intensity distribution in the far-field. The far-field is defined at $x = -7.5$ μm and is indicated with a red dashed line in Figs. S1(a,b). We present our method of calculating reflected beams parameters in Fig. S4, where the blue solid line shows the simulation results, the dashed lines indicate component Gaussian functions and the orange solid line is the sum of all Gaussian curves in a given case. In the ranges of stripe's $M_\mathrm{S}$ when LM starts and ends to be excited the beams in the far-field strongly overlap as we show in Figs. S4(a,c) where $M_\mathrm{S} = 475$ kA/m and $M_\mathrm{S} = 615$ kA/m. In these cases we need to use a sum of six Gaussian functions to precisely fit our function to the simulation results. For the stripe's $M_\mathrm{S}$ values between these regions three distinctive beams and a range with plane waves are visible in the far-field as shown in Fig. S4(b) for $M_\mathrm{S} = 550$ kA/m. Hence, we use as a fitting function the sum of four Gaussian curves only in this range of $M_\mathrm{S}$ (three to describe the beams and one to describe the plane waves).

In Fig. S5 we plot amplitudes and positions of the primary and secondary beams in the far-field as functions of stripe's $M_\mathrm{S}$. The blue colour represents parameters of the primary beams and the red colour depicts the secondary beam. Additionally in Fig. S5 we also confront the results for simulations with different frequencies, namely the dots show results for $f = 17.3$ GHz and the diamonds represent the results for $f = 17.4$ GHz. The change in frequency affects overlap between dispersion relations of the SW beam and the bilayer thus changing the excitation properties of LM in the stripe. We chose only a small change in frequency to avoid bigger change of SW wavelength which would affect the wavelength-discretization ratio in the numerical simulations. The change of frequency in the system affects the amplitudes of reflected beams as presented in Fig. S5(a). The increase of frequency to $f = 17.4$ GHz leads to increase of the primary beam's amplitude, compare blue dots and diamonds, and decrease of the secondary beam's amplitudes, compare red dots and diamonds. The same increase of frequency affect the spatial shift of the primary beam only slightly, as shown in Fig. S5(b) with blue dots and diamonds. However, the frequency increase causes substantial increase in spatial shift of the secondary beam, shown with red dots and diamonds. For $f = 17.4$ GHz the maximal shift of the secondary beam is equal to $-1.6$ μm and it is 0.35 μm bigger than spatial shift calculated for $f = 17.3$ GHz and the same value of stripe's $M_\mathrm{S}$.

### 9. *Movie S1–steady-state with a sweep over resonator's $M_\mathrm{S}$ value*

The movie. S1 represents the colourmaps of the distribution of $|m_x|$ at frequency $f = 17.4$ GHz as the dependence of the stripe's value of $M_\mathrm{S}$ similarly as displayed in Fig. 2(a,b). You can see that the distribution of $|m_x|$ is strongly affected by $M_\mathrm{S}$ and 3-6 reflected parallel laterally shifted beams can be seen depending on the $M_\mathrm{S}$ value.

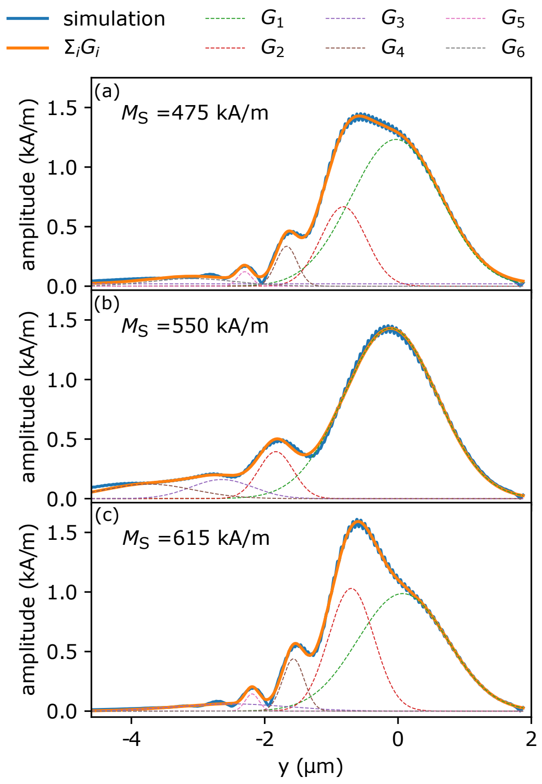


FIG. S4. Calculating parameters of the reflected beams by fitting Gaussian curves in the far-field marked in Fig. S1 with dashed-red line. (a) Fitting a sum of six Gaussian curves to the simulation results for resonator $M_S = 475$ kA/m. (b) Fitting a sum of four Gaussian curves to the simulation results for resonator $M_S = 550$ kA/m. (c) Fitting a sum of six Gaussian curves to the simulation results for resonator $M_S = 615$ kA/m.

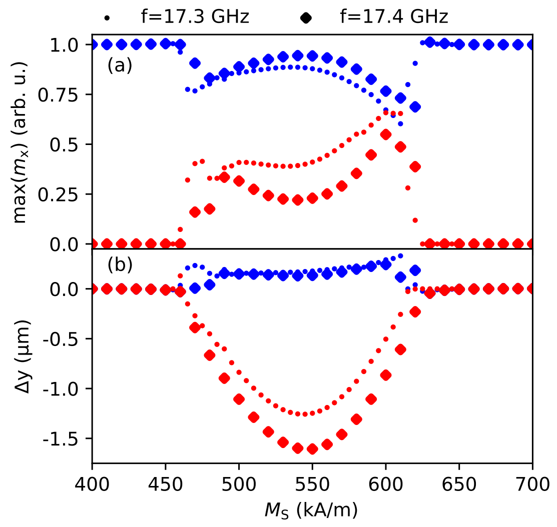


FIG. S5. Parameters of the reflected beams in simulations with different SW frequencies. (a) Amplitudes of the primary (blue) and the secondary (red) beams calculated by fitting Gaussian functions to simulation data, cf. S4, dots represent results for frequency $f = 17.3$ GHz, diamonds represent results for $f = 17.4$ GHz. (b) Positions of the primary (blue) and the secondary (red) beams calculated by fitting Gaussian functions to simulation data, symbols as represent frequencies as in (a).

### 10. Movie S2–steady-state with a sweep over frequency

The movie. S2 depicts the colourmaps of the distribution of $|m_x|$ as the dependence on the value of frequency for resonator with $M_\mathrm{S} = 550$ kA/m. We assume frequencies in the range from 17.0 to 18.0 GHz. Although, it is a narrow range, we can see that $m_x$ distribution changes significantly. While entering the resonance multiple reflected beams emerges and than again disappears. It is very similar result as for the sweep over resonator's $M_\mathrm{S}$.

### 11. Movie S3–dispersion relation dependence on resonator's $M_\mathrm{S}$

The movie. S3 depicts how the dispersion relation in the stripe depends on the value of stripe's $M_\mathrm{S}$. For the values of $M_\mathrm{S}$ from 420 kA/m to 650 kA/m we can see the crossing at $f = 17.4$ GHz and $k_y = -53.5\ \frac{\mathrm{rad}}{\mu\mathrm{m}}$ of the dispersion relation plotted for the stripe (colourmap in the background) and the dispersion plotted for SWs propagating in the layer with $\varphi = 45°$ (bold black line). For this particular band crossing in the dispersion for SWs in the layer, at the considered $M_\mathrm{S}$ range, the decrease in exchange energy is compensated by an increase of the magnetostatic energy. Namely, the exchange energy is proportional to $M_\mathrm{S}^{-1}$, while the magnetostatic energy is proportional to $M_\mathrm{S}$. It explains the origin of the broad range of $M_\mathrm{S}$ showed in Fig. 4 in the main part of the manuscript where the resonance condition are fulfilled.

### 12. Movies S4-S5–reflection of wavepackets for different $M_\mathrm{S}$

The movie. S4 shows the reflection of wavepacket from the resonant-stripe element in case of stripe $M_\mathrm{S} = 350$ kA/m. In Movie. S4 the antenna in simulation has width $w_a = 200$ nm. The movie. S5 shows SWs wavepacket reflection in case of stripe's $M_\mathrm{S} = 550$ kA/m. Comparing the results of wavepacket simulations for cases $M_\mathrm{S} = 350$ kA/m and $M_\mathrm{S} = 550$ kA/m it is evident that for latter the excitation of the SWs in the stripe is much more efficient. Without the constant SWs pumping by the SWs beam we can see propagation of a mode in the stripe as an obliquely bouncing between stripe's edges and reemition of SWs back to the layer clearly. Interestingly, in $M_\mathrm{S} = 350$ kA/m stripe case we still are able to see excitation of a mode in the stripe and the SWs reemition, however with much smaller magnitude comparing to the case with $M_\mathrm{S} = 550$ kA/m stripe. We explain this particular result by pointing that a SW wavepacket contains a range of frequencies described by a gaussian curve in our case centred at $f_0 = 17.4$ GHz with FWHM $\approx 2$ GHz. It means that the part of wavepacket spectrum still overlaps with the frequencies of resonant-stripe element's modes. This effect has small magnitude and is therefore virtually invisible in simulation results with continuous SW beam excitation.


[1] A. Vansteenkiste, J. Leliaert, M. Dvornik, M. Helsen, F. Garcia-Sanchez, and B. Van Waeyenberge, The design and verification of mumax3, AIP Adv. **4**, 107133 (2014).

[2] N. Whitehead, S. Horsley, T. Philbin, and V. Kruglyak, Graded index lenses for spin wave steering, Phy. Rev. B **100**, 094404 (2019).

[3] B. Kalinikos and A. Slavin, Theory of dipole-exchange spin wave spectrum for ferromagnetic films with mixed exchange boundary conditions, Journal of Physics C: Solid State Physics **19**, 7013 (1986).

[4] T. Tamir and H. Bertoni, Lateral displacement of optical beams at multilayered and periodic structures, J. Opt. Soc. A **61**, 1397 (1971).
